# Supplementary material for: Use of insecticide treated nets in children under five and children of school age in Nigeria: Evidence from a secondary data analysis of demographic health survey
Source: PLoS One. 2022 Sep 29;17(9):e0274160. doi: 10.1371/journal.pone.0274160 (PMC9521839; doi:10.1371/journal.pone.0274160)
Supplement: S1 File — Factors associated with ITN use in CU5 and children of school age. (DOCX) [file pone.0274160.s001.docx]

| **Variables** | **Children Under five** | | **School age children** | |
| --- | --- | --- | --- | --- |
|  | **Crude odds ratio** | **95% CI** | **Crude odds ratio** | **95% CI** |
| **Sex of head of household** |  |  |  |  |
| Male | 1.00 |  | 1.00 |  |
| Female | 1.03 | 0.88-1.21 | 1.22 | 1.08-1.38 |
| **Household own radio** |  |  |  |  |
| No | 1.00 |  | 1.00 |  |
| Yes | 0.80 | 0.73-0.88 | 0.95 | 0.88-1.04 |
| **Household own TV** |  |  |  |  |
| No | 1.00 |  | 1.00 |  |
| **Yes** | 0.67 | 0.60-0.75 | 0.88 | 0.80-0.97 |
| **When ITN was obtained** |  |  |  |  |
| Less than one year | 1.00 |  | 1.00 |  |
| 1-3 years | 1.11 | 0.46-2.70 | 0.79 | 0.34-1.89 |
| More than 3 years | 0.41 | 0.15-1.09 | 0.69 | 0.28-1.67 |
| **Number of Household members** |  |  |  |  |
| 1-3 persons | 1.00 |  | 1.00 |  |
| 4-6 persons | 0.59 | 0.49-0.69 | 0.82 | 0.70-0.94 |
| 7-9 persons | 0.41 | 0.35-0.49 | 0.61 | 0.52-0.71 |
| >9 persons | 0.32 | 0.26-0.38 | 0.40 | 0.34-0.48 |
| **Wealth quintiles** |  |  |  |  |
| Poorest | 1.00 |  | 1.00 |  |
| Poorer | 0.94 | 0.79-1.11 | 1.09 | 0.96-1.23 |
| Middle | 0.87 | 0.75-1.01 | 1.04 | 0.91-1.19 |
| Richer | 0.66 | 0.57-0.78 | 0.88 | 0.77-1.01 |
| Richest | 0.58 | 0.49-0.70 | 0.91 | 0.77-1.08 |
| **Residence** |  |  |  |  |
| Urban | 1.00 |  | 1.00 |  |
| Rural | 1.25 | 1.11-1.41 | 1.08 | 0.98-1.20 |
| **Malaria endemicity** |  |  |  |  |
| Hypoendemic | 1.00 |  | 1.00 |  |
| Mesoendemic | 2.28 | 1.77-2.95 | 2.18 | 1.73-2.73 |
| Hyperendemic | 14.86 | 9.87-22.38 | 4.79 | 3.54-6.49 |

**Table 3: Factors associated with ITN use in CU5 and children of school age.**
