# Supplementary material for: Use of insecticide treated nets in children under five and children of school age in Nigeria: Evidence from a secondary data analysis of demographic health survey
Source: PLoS One. 2022 Sep 29;17(9):e0274160. doi: 10.1371/journal.pone.0274160 (PMC9521839; doi:10.1371/journal.pone.0274160)
Supplement: Attachment — Submitted filename: PONE-D-22-02975.docx [file pone.0274160.s002.docx]

**EDITOR’S COMMENTS**

Dear Authors,

The manuscript requires major revision. The authors should critically attend all the comments raised by the reviewers. The multivariate analysis needs to be re-examined. Also, the conclusion should be rewritten to capture the findings of the study.

FIRST REVIEWER

This paper examined the usage of insecticide-treated nets in children under five and school-going children in Nigeria using a secondary analysis of demographic and health survey data. Children are important aspect of malaria elimination program across the world and this paper presents some concerns that the authors need to addressed.

Major comments

   1. As the world moves toward elimination, it would bring more valuable information to look into how stratification of malaria prevalence among children at sub-national level would look like under hypo, meso, and hyper endemic regions and how the usages of ITN net were distributed in such stratification.
   2. What is the mechanism that could link the sex of the child being female and using ITN in a household? On the other hand, gender of the head of the household is not associated with the ITN use, While sex of the child is of little value for informing policy, I would suggest the authors to use a valid theoretical framework or literature to select variables for the model.
   3. The multivariate model showed that malaria endemicity is a predictor  for the ITN usage. NCMP usually puts much efforts to bring down the incidence and prevalence in malaria endemic regions, hence this finding suggested that interventions to increase the usage of ITN are working well. Please discuss this point in the paper.
   4. The conclusion of “increase availability and access to ITN has resulted … “ is not supported by the findings in the paper. There is no results in the paper showing that the ITN availability or access to ITN increases.

Minor comments
   1. The introduction section can be shortened by moving paragraphs between lines 83 and 110 to the methods  section.
   2. Table 3: put 1 or indicate as “reference” in the reference categories, rather than indicating with a symbol.(less...)

SECOND REVIEWER

Major comments:
1.     Why it is called retrospective cross-sectional study?
2.     Result session:
There were 32087 U5C in table 1, but slept under ITN (16,671) and not slept (5,769).
There were 54692 children of school age in table 1, but slept under ITN (21,690) and not slept (15,812).
Table 3 should be multivariable logistics regression and use AOR. Need to add reference group.
Please see the table for multivariable logistics regression from other international publication.
3.     What is your implication of the study for Nigeria? What is the different information provided by your analysis compared to NDHS findings?
4.     Conclusion should provide the specific input to program.
