# Supplementary material for: Use of insecticide treated nets in children under five and children of school age in Nigeria: Evidence from a secondary data analysis of demographic health survey
Source: PLoS One. 2022 Sep 29;17(9):e0274160. doi: 10.1371/journal.pone.0274160 (PMC9521839; doi:10.1371/journal.pone.0274160)
Supplement: Attachment — Submitted filename: Response to reviewers.docx [file pone.0274160.s003.docx]

21^st^ April 2022

**Manuscript Reference#:** PONE-D-22-02975

**Title:** Use of insecticide treated nets in children under five and children of school age in Nigeria: evidence from a secondary data analysis of demographic health survey

RE: Revised manuscript submission and response to reviewer’s comments

Dear Editor,

This letter is in reference to your email dated April 5, 2022, with reviewers’ comments. We are very much delighted that the manuscript is potentially acceptable for publication in PLOS ONE once the revisions are made.

We appreciate the reviewers’ insightful comments which significantly improved the manuscript. In the current form the manuscript would make valuable contribution to the literature on this increasingly important topic. 

Please find for your kind consideration the following:

1) A section-by-section response to the comments and suggestions of the reviewers (below).
2) The revised manuscript, provided as a marked-up copy and a clean copy.

We hope the changes made on the manuscript meet with your favourable consideration. Please do not hesitate to get in touch if you require any further information. 

Chinazo Ujuju 
Corresponding Author 

**Response to Reports**

Reviewer #1:

Major comments

   1. As the world moves toward elimination, it would bring more valuable information to look into how stratification of malaria prevalence among children at sub-national level would look like under hypo, meso, and hyper endemic regions and how the usages of ITN net were distributed in such stratification.
**Response**: Thank you very much for this vital comment. The manuscript presents usage of ITN at sub national level both by geopolitical zone and state level. The use of ITN in hypo, meso and hyper endemic areas is also presented.

   2. What is the mechanism that could link the sex of the child being female and using ITN in a household? On the other hand, gender of the head of the household is not associated with the ITN use, While sex of the child is of little value for informing policy, I would suggest the authors to use a valid theoretical framework or literature to select variables for the model.

**Response**: Previous studies included sex as a key socio-demographic factor for health-related outcomes. Similarly, we selected sex of household head and child to examine the association with ITN use. As suggested, literatures that were considered in selecting the variables has been indicated.

The following literatures included sex as one of the determinants of ITN use in children:

- Olapeju B, Choiriyyah I, Lynch M, Acosta A, Blaufuss S, Filemyr E, et al. Age and gender trends in insecticide-treated net use in sub-Saharan Africa: a multi-country analysis. Malar J [Internet]. 2018 Dec [cited 2021 Nov 10];17(1):423. Available from: <https://malariajournal.biomedcentral.com/articles/10.1186/s12936-018-2575-z>
- Nkoka O, Chipeta MS, Chuang Y-C, Fergus D, Chuang K-Y. A comparative study of the prevalence of and factors associated with insecticide-treated nets usage among children under 5 years of age in households that already own nets in Malawi. Malaria Journal [Internet]. 2019 Dec [cited 2021 Oct 8];18(1):43. Available from: https://malariajournal.biomedcentral.com/articles/10.1186/s12936-019-2667-4
- Mensah EA, Anto F. Individual and Community Factors Associated with Household Insecticide-Treated Bednet Usage in the Sunyani West District of Ghana Two Years after Mass Distribution. Journal of Environmental and Public Health. 2020;2020: 1–7. doi:10.1155/2020/7054383

3. The multivariate model showed that malaria endemicity is a predictor for the ITN usage. NCMP usually puts much efforts to bring down the incidence and prevalence in malaria endemic regions, hence this finding suggested that interventions to increase the usage of ITN are working well. Please discuss this point in the paper

**Response**: Thank you very much for this vital comment. This has been addressed in the manuscript.

   4. The conclusion of “increase availability and access to ITN has resulted … “ is not supported by the findings in the paper. There is no results in the paper showing that the ITN availability or access to ITN increases.

**Response**: The conclusion has been revised accordingly Thank you.

Minor comments
   1. The introduction section can be shortened by moving paragraphs between lines 83 and 110 to the methods section.

**Response**: The introduction section has been shortened Thank you.

   2. Table 3: put 1 or indicate as “reference” in the reference categories, rather than indicating with a symbol.(less...)

**Response**: Table has been revised accordingly Thank you.

SECOND REVIEWER

Major comments:
1.     Why it is called retrospective cross-sectional study?

Response: The demographic health survey was conducted in 2018 while secondary analysis for the manuscript was done in 2021. We adjudged the study to be retrospective. However, we have removed “retrospective” for clarity.

2.     Result session:
There were 32087 U5C in table 1, but slept under ITN (16,671) and not slept (5,769).

Response: Table 2 was based on children in household who own at least 1 ITN. This variable has been added to Table 1 and also shows number of children in household without ITN for clarity Thank you.

3. There were 54692 children of school age in table 1, but slept under ITN (21,690) and not slept (15,812).

Response: Table 2 was based on children in households who own at least 1 ITN. This variable has been added to Table 1 and also show number of children in household without ITN.

Table 3 should be multivariable logistics regression and use AOR. Need to add reference group.

Response: Reference group has been added.

Please see the table for multivariable logistics regression from other international publication.

Response: Table has been revised accordingly Thank you.

3.     What is your implication of the study for Nigeria? What is the different information provided by your analysis compared to NDHS findings?

Response: Response has been included in the manuscript Thank you.

4.     Conclusion should provide the specific input to program

Response: Conclusion revised.
