# Supplementary material for: Use of insecticide treated nets in children under five and children of school age in Nigeria: Evidence from a secondary data analysis of demographic health survey
Source: PLoS One. 2022 Sep 29;17(9):e0274160. doi: 10.1371/journal.pone.0274160 (PMC9521839; doi:10.1371/journal.pone.0274160)
Supplement: Attachment — Submitted filename: Draft Rebuttal Letter.docx [file pone.0274160.s004.docx]

REVIEWER 1

The authors have addressed my comments and concerns well and adequately. No further comments from my end.

Noted

REVIEWER 2

Major comments:

I would like to suggest to reconsider the analysis because your study objective is to determine the use of ITN in under five and children of school age in Nigeria.

We presume our analysis was done to answer the objectives of the study. The stratification of the analysis into two categories children under five and children of school age meets the objective of our study. Separating the two categories was intentional to ensure that future public health decisions can consider children under five and school aged children independently.

In the result, you mentioned that “more than 80% of CU5 and school children used ITN”. This finding magnify your result of use of ITN because you used the denominator of those who had at least 1 ITN. It cannot be reflect the whole study population of CU5 and school children in Nigeria.

Thank you for pointing out this very crucial mis-representation of the findings. The population of children under five and children of school age who slept under ITN was 74.3% and 57.8%% respectively and has been included in the result. The result also presented ITN use at state level and described states that had more than 80% ITN coverage in children under five and children above five ( as you presented above). NMEP set vector control utilization target of 80% and this has only been measured at national level using DHS. This manuscript presented state level analysis and the result described states that reached the 80% utilization target for ITN.

You need to discuss about the % of access to ITN, and use of ITN in total population, and use of ITN in those who had access. Need to compare 2 use of ITN and consider the implications of the study finding. Therefore, your study objective should focus the access and use of ITN. You cannot omit “access” indicator to improve the ITN utilization implementation in Nigeria.

Our study population is children under five and children of school age. Access to ITN is a standard malaria indicator determined at household level. Access to ITN is calculated by dividing the sum of all potential ITN users in the sample by the total number of individuals who spent the previous night in surveyed households. This is based on the assumption that 2 people can sleep under one ITN. As such a household with 4 residents will require 2 ITN. (Reference Kilian, A, H. Koenker, and L. Paintain. 2013. "Estimating population access to insecticide-treated nets from administrative data: correction factor is needed." *Malaria journal* 12(1): 259. <https://malariajournal.biomedcentral.com/articles/10.1186/1475-2875-12-259>). As the study did not focus on the entire household but on children under five and children of school age, determining household access to ITN is beyond the scope of this study.

To determine use of ITN in the study population which is children under five and children of school age, it is not appropriate to ask whether a child living in a household that do not have an ITN slept under ITN. As such the study explored use of ITN in households that have at least one ITN. That shows the child had access to ITN. This is a standard way of determining use of ITN in DHS analysis, presented in the DHS report and is available in several literatures. In addition, our literature already documents efforts by the Nigerian government to increase access. We choose to streamline the focus of this analysis such that our public health message will be clear which is the use of ITN. We have revised the manuscript further to have the introduction and discussion focus on utilization of ITN.

Minor comments:

1. Why it is called retrospective cross-sectional study?

R1: Please remove “retrospective” for the clarity. Here is the sample that used in other manuscript.

“This study analysed the secondary data from MDHS 2015-16, which is a cross-sectional study.”

Retrospective has been removed

2. Result session:

There were 32087 U5C in table 1, but slept under ITN (16,671) and not slept (5,769).

R1: Thank you for clarification. Please add total N in the table 1 and 2. I would like to suggest to remove the “Never slept” column for clear vision and eyeballing of the table.

In the tables, P value should be <0.001. No need to describe <0.0001.

Total for table 1 and 2 added, P value revised and column on never slept removed from table 2

3. There were 54692 children of school age in table 1, but slept under ITN (21,690) and not slept (15,812).

R1: OK.

Table 3 should be multivariable logistics regression and use AOR. Need to add reference group.

Reference group has been added.

Please see the table for multivariable logistics regression from other international publication.

Table has been revised accordingly

R1: Please mention multivariable logistic regression if you have adjusted the other factors. Please describe AOR instead of OR in the table 3 and in text.* is not the reference value. It is the reference group.

Generally, we describe “1” or Ref in the table. See in the below figure.

3. What is your implication of the study for Nigeria? What is the different information provided by your analysis compared to NDHS findings?

Response has been included under strength of the manuscript

4. Conclusion should provide the specific input to program

Conclusion has specific input to program and recommendation

R1: Please mention study strength and limitation instead of study limitation on line 301. It is mixed for strength and limitation.

Study strength and limitation mentioned as recommended

REVIEWER 3

The study examined the use of insecticide treated nets and its correlates among children under five and children of school age in Nigeria using the 2018 Nigeria demographic and health survey data. They attempt to address very important public health challenge, especially in sub-Sahara African countries like Nigeria. Their decision to consider both the under 5s and those aged 5-14 is commendable. Studies of this nature are relevant to policymakers and other stakeholder for informed decision making. However, I have a reservation about ignoring the hierarchical structure of the DHS data used in their binary logistic regression models presented.

The background to the study looks good.

Like any other DHS data, the Nigeria DHS data is hierarchical in nature where we have children nested within households, and household nested within clusters (i.e., communities) but the authors did not explain how they account for the hierarchical structure of the data used in this study. Assuming this was not explored during their modelling stage using multilevel (i.e., mixed effect) regression analysis, it could lead to spurious statistical significance with its associated misleading interpretations. Fortunately, we have statistical software packages that allow easy implementation of the multilevel binary logistic regression analysis. Authors are encouraged to explore this and compare the results with the single level binary regression to improve the quality of their results in the manuscript.

We have considered the suggestion and multilevel logistic regression analysis has been implemented. It’s Important to mention that the study has benefited from implementing a multilevel regression with the models defined at different levels of analysis.

Also, since the authors considered both children <5s and 5-14 years and analysed the data separately for these two (2) groups, it could be very useful to pool (i.e., combined) this data and conduct another analysis to assess the correlates for the pooled dataset. This result can then to compared to the separate analysis to inform policy decisions.

We didn’t think the pooled analysis will be of much public health importance. Children under 5 are critical age in public health and school age children are also significant especially with the increased prevalence of malaria reported in the age group. We are not aware of any significance analysis of data for under 14 children can add to public health. The suggestion is noted and will be considered in future studies.

Furthermore, the reference categories, the adjusted odds ratios, and p-values in Table 3 are not properly presented, and they should present them in a standard format. The authors should see Table 4 in the publication

https://journals.plos.org/plosone/article?id=10.1371/journal.pone.0257944 for guidance. Also, is not acceptable to present a p-value=0.000 in a manuscript. This should be written as <0.01 or <0.001 in case of p-value=0.0000.

This suggestion is noted and table 3 has been revised

In the conclusion, I struggled to link the analysis done by the authors to their conclusion that “Based on the findings of this study, increased availability and access to ITN has resulted in an increased use by children in rural areas and poor households”. Clearly, this cannot be supported with the data available. I have noted that the previous Reviewer #1 raised same query in item 4 which the authors stated that they have revised in the conclusion and yet they actually did not revise this. I encourage the authors to pay attention to all queries raised and address them to the best of their ability.

Apologies for the oversight. The conclusion has been revised

The manuscript will also benefit from some few proofreading to improve the message.

Manuscript has been reviewed and errors addressed

REVIEWER 4

Comments:

1. How collinearity test was conducted?

Variance inflation factor has been calculated to determine the extent of collinearity. Multilevel logistic regression analysis was used which ensures that correlation effect was reduced in the estimation of model parameters.

2. How were the variables selected in the final model?

The variables were selected based on association in the bivariate analysis while the model applied the forward method in variable inclusion in the multilevel logistic regression.

3. Did the authors adjust for the survey design and cluster effect? Please explain.

The design of the DHS survey has adjusted for this effect. Also, the use of multilevel regression analysis ensures that the nested structure of DHS data is taking into effect in the analysis and the variability of the data at different levels of sample selections was measure and presented in table 3.

4. In table 3, both crude and adjusted odds ratio should be shown.

Given that we have now conducted multilevel logic regression, stratified the model and considered different levels of sampling in the analysis we presume that presenting crude odds ratio may not be necessary in the analysis.
